# The role of cardiac glycosides in influencing breast cancer cell proliferation

**DOI:** 10.1186/1753-6561-6-S4-P1

**Published:** 2012-07-09

**Authors:** KD Quinlan, MB Owens, ADK Hill, AM Hopkins

**Affiliations:** 1 Department of Surgery, Royal College of Surgeons in Ireland, Education & Research Centre, Beaumont Hospital, Dublin, Ireland

## Introduction

Cardiac Glycosides (CGs) are commonly used to treat congestive heart failure. CGs inhibit the Sodium Potassium ATPase (Na+/K+ ATPase) pump. Interestingly, CGs have been suggested to inhibit proliferation and migration of breast cancer cells. A pool of non-pumping Na+/K+ ATPase reportedly localizes in specific membrane organelles, caveolae, by interacting with the structural protein caveolin-1. It has been postulated that Na+/K+ ATPase forms a complex with caveolin-1 and the tyrosine kinase Src, and that binding of CGs to Na+/K+ ATPase activates Src-dependent signalling cascades. In this project we explored whether CGs reduce proliferation in breast cancer cells and whether these effects might involve Src and ERK (Extracellular-signal-Regulated Kinases).

## Methods

MDA-MB 231 cells [highly-invasive breast cancer cells] were transfected with siRNA to knock down caveolin-1 expression. MDA-MB-231 cells in which caveolin-1 was knocked down and MCF 10A cells (non-invasive breast cancer cells) were treated with CGs and subjected to MTT proliferation assays. MCF 7 cells (weakly-invasive breast cancer cells) were treated with the Src inhibitor PP2 in the presence or absence of CGs and analysed by western blotting for phosphorylated Src and phosphorylated ERK.

## Results

High dose CGs (Digoxin>150nM, Ouabain>10nM, Oleandrin>100nM) reduced proliferation in MCF 10A cells over 72 hrs. Caveolin-1 was successfully knocked-down in MDA-MB 231 cells, but this did not appear to abrogate the anti-proliferative effects of CGs. Early results suggest that phospho-Src and phospho-ERK expression were increased in MCF 7 cells treated with Digoxin. Interestingly, this was not abrogated by pre-treatment with the Src inhibitor PP2.

## Conclusions

This project has demonstrated that CGs exert anti-proliferative effects on a range of breast cancer cell lines. Early results suggest that the effects of CGs may not be directly linked to Src and ERK signalling. Ongoing work is determining whether caveolin-1 knockdown alters the anti-proliferative response to CG treatment. We suggest that further exploration of the mechanisms whereby CGs inhibit proliferation may reveal potential uses for CGs as anti-cancer drugs in the future.

